# In Situ Phase Separation Strategy to Construct Zinc Oxide Dots-Modified Vanadium Nitride Flower-like Heterojunctions as an Efficient Sulfur Nanoreactor for Lithium-Sulfur Batteries

**DOI:** 10.3390/ma18112639

**Published:** 2025-06-04

**Authors:** Ningning Chen, Wei Zhou, Minzhe Chen, Ke Yuan, Haofeng Zuo, Aocheng Wang, Dengke Zhao, Nan Wang, Ligui Li

**Affiliations:** 1 New Energy Research Institute, College of Environment and Energy, South China University of Technology, Guangzhou 510006, China; 18909544258@163.com (N.C.); 17827099521@163.com (W.Z.); chenminzhe321@163.com (M.C.); yuan960948355ke@163.com (K.Y.); haofeng_zuo@163.com (H.Z.); wangac0713@163.com (A.W.); 2 School of Materials Science and Engineering, Henan Normal University, Xinxiang 453007, China; 3 Siyuan Laboratory, Guangzhou Key Laboratory of Vacuum Coating Technologies and New Energy Materials, Guangdong Provincial Engineering Technology Research Center of Vacuum Coating Technologies and New Energy Materials, Guangdong Provincial Key Laboratory of Nanophotonic Manipulation, Department of Physics, Jinan University, Guangzhou 510632, China; nanwang@email.jnu.edu.cn

**Keywords:** lithium-sulfur battery, cathode, built-in electric field, heterojunction

## Abstract

Exploring advanced sulfur cathode materials is important for the development of lithium-sulfur batteries (LSBs), but they still present challenges. Herein, zinc oxide dots-modified vanadium nitride flower-like heterojunctions (Zn-QDs-VN) as sulfur hosts are prepared by a phase separation strategy. Characterizations confirm that the flower structure with high specific surface area and pores improves active site exposure and electron/mass transfer. In situ phase separation enriches the Zn-QDs-VN interface, addressing the issues of uneven distribution and interface reduction of Zn-QDs-VN. Further theoretical computations reveal that ZnO-QDs-VN with optimized intermediate spin states can constitute a stable LiS* bond sequence, which can conspicuously facilitate the adsorption and conversion of LiPSs and reduce the battery reaction energy barrier. Therefore, the ZnO-QDs-VN@S cathode shows a high initial specific capacity of 1109.6 mAh g^−1^ at 1.0 C and long cycle stability (maintaining 984.2 mAh g^−1^ after 500 cycles). Under high S loading (8.5 mg cm^−2^) and lean electrolyte conditions (E/S = 6.5 μL mg^−1^), it also exhibits a high initial area capacity (10.26 mAh cm^−2^) at 0.2 C. The interfacial synergistic effect accelerates the adsorption and conversion of LiPSs and reduces the energy barriers in cell reactions. The study provides a new method for designing heterojunctions to achieve high-performance LSBs.

## 1. Introduction

Lithium-sulfur batteries (LSBs) are considered one of the new energy systems with promising applications due to their attractive energy density (2600 Wh kg^−1^) and high theoretical capacity (1675 mAh g^−1^) [1,2]. However, the inherent drawbacks of the cathode, such as rapid volume expansion, the notorious shuttle effect of lithium polysulfide (LiPSs), and sluggish kinetics of S-involved reaction, greatly limit large-scale applications of LSBs [3,4]. To overcome these challenges, great effort has been devoted to exploring advanced sulfur host materials [5]. Among them, metal oxides (MOs such as ZnO, Co_3_O_4_, and MnO_2_) that have good polarity and can strongly absorb LiPSs by chemical binding to suppress the shuttle effect are considered potential candidates [6,7,8]. Unfortunately, the strong adsorption capacity and poor conductivity of MOs often lead to sluggish electrochemical conversion of LiPSs, thus greatly restricting the performance of LSBs [9,10].

In recent years, many studies have shown that vanadium nitride (VN) with metallic properties exhibits exceptional catalytic performance for sulfur redox reactions due to its excellent electrical conductivity and suitable d-band structures [11,12]. For example, Yang et al. prepared an N-doped porous graphitic carbon-supported VN as a superior electrocatalytic cathode host for LSBs with a high specific capacity of 1442 mAh g^−1^ at 0.1 C [13]. Moreover, VNs such as CC/VN/Co@NCNTs [14] and VN/NG [15] have also been developed as electrocatalysts for enhancing LSB performance. Therefore, reasonable coupling with VN to build heterostructures may be an effective way to overcome the shortcomings of MOs. Moreover, due to the difference in work function (WF) between VN and MOs, electrons can be spontaneously transferred at the interface to form a built-in electric field (BIEF), thus helping to further optimize each other’s electronic structure to balance the adsorption capacity and catalytic activity toward LiPSs [16,17]. In addition to improving intrinsic activity, the method of fully expose accessible active sites is equally important for overall performance. For heterojunctions, the main activity sites with high performance are located at the interface in general [18]. Nevertheless, the most common method of preparing heterostructures is the ex situ approach, where each component or precursor is synthesized separately and then combined, often resulting in uneven distributions, reduced heterointerfaces, and, consequently, weakened interactions. How to construct heterojunctions with tight interfaces remains a significant challenge.

In this work, a zinc oxide dots-modified VN flower-like heterojunction (Zn-QDs-VN) is prepared by a self-template limited and phase separation strategy as an efficient sulfur nanoreactor. Characterizations show that ZnO-QDs with a nanosize (<10 nm) are encapsulated into porous flower-shaped nanoreactors to enrich the interfaces, thus strongly enhancing the interaction. Moreover, the partial Zn volatilization in the synthesis process significantly improved the specific surface area and pore structure of samples, which provided more space to store the S nanoparticles, expose active sites, and improve the electron/mass transfer. Further theoretical calculations indicate that ZnO-QDs-VN with optimized intermediate spin states can form stable LiS* bond sequences, which can significantly promote the adsorption and conversion of LiPSs and reduce the energy barrier in battery reactions. Based on the above advantages, the ZnO-QDs-VN@S cathode exhibits a high initial specific capacity of 960.8 mAh g^−1^ at 3.0 C and can maintain a specific capacity of 687.9 mAh g^−1^ after 500 cycles. Moreover, even under harsh conditions (sulfur loading of 8.5 mg cm^−2^ and E/S = 6.5 μL mg^−1^), it also exhibits an extremely high initial area capacity (10.258 mAh cm^−2^), and the capacity retention (CR) is 60.31% after 100 cycles.

## 2. Experimental Section

### 2.1. Materials

All chemical reagents purchased for unprocessed use were of analytical grade. Ammonium metavanadate (NH_4_VO_3_) was bought from Shanghai Maclin Biochemical Co., Ltd. (Shanghai, China). Zinc nitrate hexahydrate (Zn(NO_3_)_2_·6H_2_O) was purchased from Guangzhou Qian Hui Glass Instrument Co., Ltd. (Guangzhou, China). The water used in this experiment was pure (99.7%).

#### 2.1.1. Preparation of Zn_3_(OH)_2_(V_2_O_7_)(H_2_O)_2_

First, 0.421 g of NH_4_VO_3_ was dispersed in 20 mL of distilled water at 80 °C to form solution A. The Zn(NO_3_)_2_·6H_2_O (1.070 g) was dissolved in 20 mL of distilled water and stirred at room temperature for 20 min to form solution B. Then, solution A was slowly poured into solution B with stirring for 10 h at room temperature to obtain a uniform orange solution. Finally, the Zn_3_(OH)_2_(V_2_O_7_)(H_2_O)_2_ precursor was collected and dried at 60 °C overnight.

#### 2.1.2. Preparation of Porous ZnO-QDs-VN and Other Specimens for Comparison

ZnO-QDs-VN was prepared through the calcination process. The obtained Zn_3_ (OH)_2_(V_2_O_7_)(H_2_O)_2_ and Melamine (C_3_H_6_N_6_) (weight ratio 1:10) were placed in two different locations in a porcelain vessel, with C_3_H_6_N_6_ positioned upstream of the furnace. The mixture was then calcined for 0.5 h at 750 °C in an argon atmosphere at a slope rate of 3 °C min^−1^ and denoted as ZnO-QDs-VN. However, the VN nanosheets were prepared by etching ZnO-QDs-VN in 0.1 M HCL solution for 24 h, and the VN nanosheets were filtered and dried overnight at 60 °C. The ZnO was prepared by calcining zinc acetate at 450 °C in air for 2 h.

#### 2.1.3. Preparation of ZnO-QDs-VN@S, VN@S and ZnO@S

The ZnO-QDs-VN@S composites were obtained by mixing sulfur powder and as-prepared ZnO-QDs-VN (weight ratio 7:3) and heating at 155 °C (ramp rate of 2 °C min^−1^) for 12 h under an argon atmosphere, followed by further heating to 200 °C for 1 h to remove excess sulfur. VN@S and ZnO@S composites were synthesized in the same manner.

#### 2.1.4. Analysis of Synthetic Mechanism

The synthesis mechanism is based on the synergistic effect of precursor coordination assembly and melamine, forming the target structure through high-temperature in situ phase separation.

Firstly, after dissolving ammonium metavanadate and zinc nitrate, Zn^2^⁺ forms a Zn-V-O-N-C composite coordination network with VO₄^3^⁻ and amino groups of melamine. Elemental molecular-level uniform dispersion is achieved through stirring and drying. Secondly, in argon at 750 °C, melamine decomposes to generate reducing gases (such as NH₃) and N^3^⁻, which reduce V⁵⁺ to V^3^⁺ and promote VN formation; zinc nitrate decomposes to form ZnO, while carbon residues from melamine serve as 2D growth templates. Subsequently, the higher bond energy of V-N compared to Zn-O drives preferential formation of VN nanosheets, while ZnO desorbs as quantum dots due to lower bond energy; carbon skeletons from melamine decomposition restrict ZnO grain growth and anchor them onto VN surfaces, forming heterojunction composite structures. Finally, argon protection prevents excessive oxidation, and 750 °C balances reaction kinetics and grain size control, ultimately achieving in situ dispersion and doping of ZnO quantum dots on VN nanosheets.

#### 2.1.5. Preparation of Li_2_S_6_ Solution for the Adsorption Experiments

The Li_2_S and S powder (molar ratio 5:1) were dissolved in tetrahydrofuran (THF) solution to give 0.2 M Li_2_S_6_/THF solution. Subsequently, the mixture was put in DOL/DME (volume ratio 1:1) solution and stirred for 12 h at 55 °C in a glove box. ZnO-QDs-VN and VN were added to 6 mL of the above-prepared solution, which was allowed to stand for some time to observe the color, evaluate the adsorption effect of the material on LiPSs, and further analyze the content of Li_2_S_6_ by UV-vis.

#### 2.1.6. Lithium Sulfide Deposition and Dissolution Experiments

The Li_2_S and S powder (molar ratio 7:1) were dissolved in ethylene glycol dimethyl ether (TEG) solution to obtain 0.2 M Li_2_S_8_/TEG as cathode electrolyte and conventional LSBs as anode electrolyte. ZnO-QDs-VN@S, VN@S was applied to the carbon paper as the anode of the battery, and the lithium sheet was assembled into the battery as the cathode. Lithium sulfide deposition experiments were performed with a constant current of 0.112 mA, then kept at 2.05 V with a constant voltage discharge until the current was below 10^−5^ mA to obtain an i–t curve. For lithium sulfide dissolution experiments, the prepared battery was discharged to 1.7 V at 0.112 mA and then charged at 2.24 V constant voltage until the current was below 10^−5^ mA to obtain an i–t curve.

#### 2.1.7. Voltammograms of Symmetric Cells

The prepared ZnO-QDS-VN and VN were used as the working electrodes of the symmetric cell, and the Li_2_S_6_ solution of 20 μL (0.2 M) was used as the electrolyte of the symmetric cell. The CV or EIS of the battery was then obtained within a certain scanning rate and voltage range (0.8–−0.8 V). This study used the CHI 660D electrochemical workstation (Shanghai Chen Hua Co., Ltd., Shanghai, China) to test.

#### 2.1.8. Constant Current Titration Interval (GITT) Test

In this study, GITT under the first circle of ZnO-QDs-VN@S and VN@S was performed under the test condition of 0.1 C for charging and discharging for 10 min, and then allowed to stand for 1 h. Then, the charge/discharge curves of ZnO-QDs-VN@S and VN @S were analyzed to calculate the internal resistance of LSBs at different charge–discharge stages of ZnO-QDs-VN@S and VN@S.

#### 2.1.9. Electrochemical Performance Test of the Coin Battery

Active materials (for example, ZnO-QDs-VN@S, ZnO, and VN), Super P, and PVDF were mixed with N-methyl-pyrrolidone (NMP) at a molar ratio (7:2:1) to form a uniform dispersion for 6 h. Then, the mixture was coated on carbon-coated aluminum foil (with a thickness of 300 μm) and dried overnight at 60 °C. After drying, the foil was cut into circular thin slices with an area of 1.131 cm^2^ using a slicer. Weight loading: The loading of the active substance was 1.2 mg cm^−2^ (calculated by weighing). Electrode conductivity: The conductivity of the electrode sheet was measured to be 7 S/cm by the four-probe method. The prepared active materials as the anode, lithium metal foil as the cathode, and commercial polypropylene (PP) as the membrane were used to assemble CR2032 coin batteries.

The electrolyte consisted of 1 M LiTFSI dissolved in a binary solvent of 1,3-dioxopentane (DOL)/ethylene glycol diformaldehyde (DME) (*v*/*v* is 1:1), and 2 wt% lithium nitrate (LiNO_3_) was added as the electrolyte additive. Current density: All test current densities were normalized by electrode area (cm^2^), with the unit being mA cm^−2^ (for example, in constant current charge and discharge tests, a current density of 1 mA cm^−2^ corresponds to a total current of 1.131 mA).

### 2.2. Material Characterization

Scanning electron microscopy (SEM, Tescan Mira LMS, Taiciken Trading (Shanghai) Co., Ltd., Shanghai, China) and transmission electron microscopy (TEM, JEM-ARM300F, JEOL, Tokyo, Japan) were used to record the morphology of the as-prepared materials. The structure and crystal structure information of the as-prepared materials were analyzed by X-ray diffractometer (XRD, Bruker D8 ADVANCE, Bruker AXS GmbH, Karlsruhe, Germany) and X-ray photoelectron spectroscopy (XPS, PHI X-tool, Chigasaki, Kanagawa, Japan). The pore structure and surface properties of the as-prepared materials were characterized by using a nitrogen adsorption–desorption instrument (Quantachrome Instruments. Boynton Beach, FL, USA). Thermogravimetry (TGA, Mettler-Toledo Group. Zurich, Switzerland) to determine the sulfur mass fraction of the as-prepared materials in N_2_. The adsorption properties of lithium polysulfide were tested using a UV–vis spectrophotometer (UV2600-2200 CH, Shimadzu Corporation, Kyoto, Japan).

## 3. Results and Discussion

### 3.1. Physical Characterization and Component Analysis of ZnO-QDs-VN Electrocatalyst

**Figure 1:**
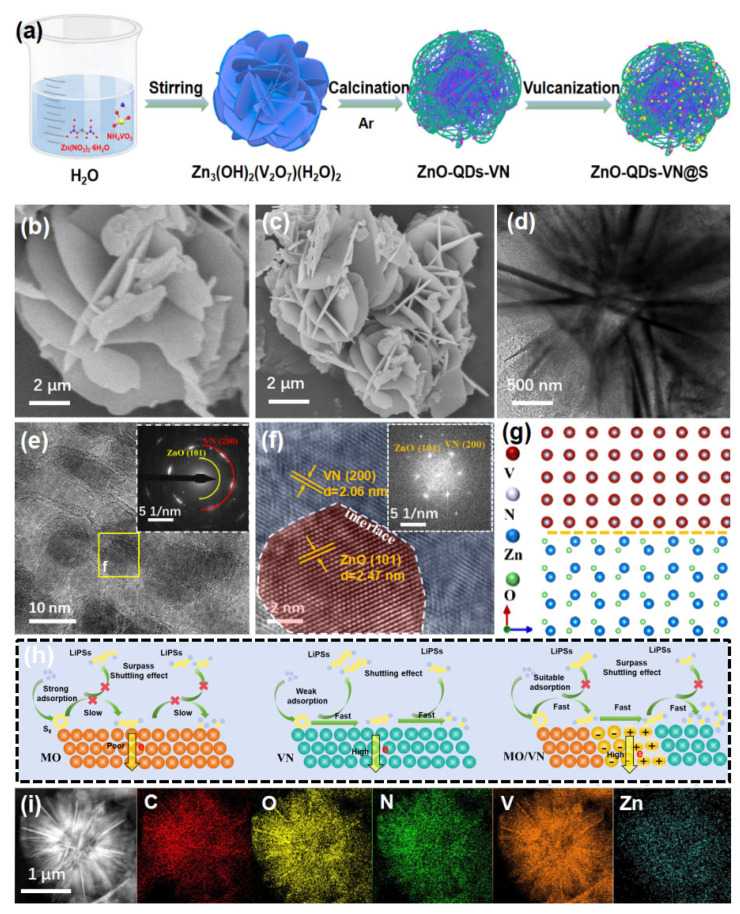
(**a**) Schematic diagram of the synthesis of ZnO-QDs-VN@S (The red dots represent zinc oxide quantum dots, and the yellow dots denote sulfur). (**b**,**c**) SEM images of Zn_3_(OH)_2_(V_2_O_7_)(H_2_O)_2_ and ZnO-QDs-VN. (**d–f**) TEM and HRTEM images of ZnO-QDs-VN (Inset: the SAED pattern and FFT plots). (**g**) Model of ZnO/VN heterojunction. (**h**) Schematic diagram of the activity enhancement mechanism (the orange spheres represent zinc oxide, and the cyan spheres denote vanadium nitride). (**i**) Element mappings (C, O, N, V, Zn) of ZnO-QDs-VN.

Figure 1a shows the synthesis process for the ZnO-QDs-VN@S, where the Zn_3_ (OH)_2_(V_2_O_7_)(H_2_O)_2_ nanoflowers are obtained via the coprecipitation at ambient temperature; the corresponding SEM image is displayed in Figure 1b. Subsequently, the ZnO-QDs-VN nanosheets with enriched pore size were prepared by a one-step nitriding process. In the SEM images (Figure 1c), ZnO-QDs-VN can still maintain the nanoflower-like morphology well. As control samples, VN samples were also prepared by etching ZnO-QDs-VN with acid. Due to the leaching of ZnO, the surface of VN becomes rough (Figure S1a). Finally, the ZnO-QDs-VN@S was prepared by the conventional melt diffusion method. In Figure S1b, one can find that the morphology of ZnO-QDs-VN is almost changed after loading of S. In addition, no obvious sulfur particles can be observed on the surface, indicating that S particles are confined within a 3D flower-like structure. The information of phase was analyzed by the XRD pattern (Figure S2a), where ZnO-QDs-VN exhibited five distinct diffraction peaks at 37.9°, 44.4°, 64.5°, 77.2°, and 81.4°, corresponding to the (111), (200), (220), (311), and (222) planes of VN (PDF# 73-2038) [19,20]. Moreover, four other peaks at 30.1°, 35.4°, 46.2°, and 54.5° were also observed and belonged to the (100), (101), (102), and (110) planes of ZnO (PDF # 36-1451) [21], confirming that VN and ZnO were successfully synthesized in the samples. Furthermore, after S loading, the XRD pattern of ZnO-QDs-VN@S (Figure S2b) showed a series of intense sulfur diffraction peaks (PDF#08-0247), suggesting that sulfur was successfully encapsulated in the ZnO-QDs-VN host [3].

TEM images (Figure 1d and Figure S3) further demonstrated that ZnO-QDs-VN had a nanoflower-like morphology, and the uniform dispersion of ZnO quantum dots on the surface of VN nanosheets could be clearly observed in the images. The HRTEM images (Figure 1e,f) displayed clear lattice fringes with a spacing of 0.206 and 0.247 nm, corresponding to the (200) crystal plane of VN and the (101) crystal plane of ZnO (JCPDS 36-1451), respectively. Furthermore, Rietveld refinement of the XRD data was carried out through fitting calculations (Figure S4). The results show that the grain sizes of the ZnO phase and VN phase of the ZnO-ODS-VN composite material were 10.32 nm and 26.94 nm, respectively, which is consistent with the HRTEM observation results. The SAED pattern and FFT plots (Figure 1e,f inset) further support the results; the obvious diffraction rings and spots of VN (200) and ZnO (101) can be observed [19,21]. In Figure 1f, one can see the clear interface between VN and ZnO-QDs, which suggests the formation of a heterojunction in ZnO-QDs-VN (the corresponding model is shown in Figure 1g). Compared to single ZnO, the ZnO/VN heterojunction can fully combine each other’ s strengths to enhance the catalytic and adsorption capacity toward LiPSs, thereby effectively inhibiting the shuttling effects and accelerating the cell reaction kinetics to improve the performance of LSBs (Figure 1h). Moreover, the mapping images (Figure 1i) show that the elements of C, O, N, V, and Zn are uniformly dispersed throughout the sample surface.

The specific surface area (SAA) and pore size distribution were characterized by BET measurements. The N_2_ adsorption/desorption plots exhibited the characteristics of a type IV isotherm, with a distinct hysteresis loop evident in the pressure data, indicating the coexistence of micropores and mesopores within the materials [22]. The ZnO-QDs-VN showed a high SAA of 150.453 m^2^ g^−1^. Furthermore, the pore size distribution plots indicated that ZnO-QDs-VN exhibits a high pore volume of 0.489 cm^3^ g^−1^ and a substantial pore size with an average diameter of 8.647 nm (Figure S5a). In Figure S5b, VN exhibits the same SAA, pore volume, and average pore size as ZnO-QDs-VN. The large SAA, high pore volume, and suitable pore size cannot only help to provide more space to store active S species, but also enhance the mass transfer. The N_2_ adsorption/desorption and pore size distribution plots (Figure S5c) of ZnO-QDs-VN@S confirm this view, where the SAA and pore volume decrease rapidly compared to ZnO-QDs-VN. This phenomenon is primarily attributed to the melting of S into the pore structure, which effectively obstructs the pore. Furthermore, thermogravimetric analysis (TGA) (Figure S6) corroborates the exceptional sulfur-carrying capacity of ZnO-QDs-VN, with an S loading of up to 69%, which ensures an improvement in the energy density of LSBs.

**Figure 2:**
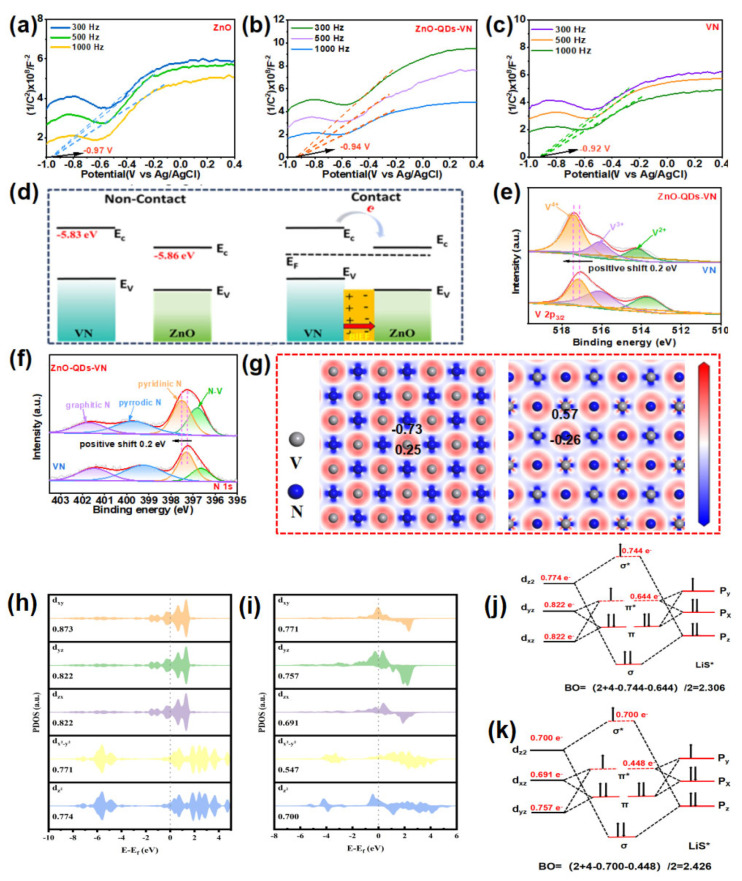
Mott–Schottky plots of (**a**) ZnO, (**b**) ZnO-QDs-VN, and (**c**) VN at different frequencies. (**d**) Schematic diagram of band structure before and after contact between VN and ZnO. High-resolution XPS spectra of (**e**) V 2 p_3/2_, (**f**) N 1s. (**g**) Schematic diagram of differential charge density of VN, ZnO-QDs-VN. The PDOS of (**h**) VN, (**i**) ZnO-QDs-VN. Schematic diagram of the MO showing the coupling of the LiS* reaction intermediate with the V sites on (**j**) VN and (**k**) ZnO-QDs-VN.

To analyze the electron interaction between ZnO quantum dots (QDs) and vanadium nitride (VN), Mott–Schottky (M-S) plots (Figure 2a–c) were initially conducted. The positive slopes observed indicate that both ZnO and VN are n-type semiconductors [10]. Consequently, the formation of a heterojunction in ZnO-QDs-VN is an n-n type junction. From the M–S plots, the flat band potentials (E_FB_) of the fitted plots of ZnO-QDs-VN, VN, and ZnO were evaluated and determined to be −0.94, −0.92, and −0.97 V (vs. Ag/AgCl), respectively. The positions of the conductive bands could be calculated as −5.85 eV, −5.82 eV, and −5.86 eV (vs. Vac.) from these values. It is evident that the conductive band of ZnO-QDs-VN is between VN and ZnO, which means electrons at the interface between ZnO-QDs and VN spontaneously transfer to form a BIEF (Figure 2d), thus helping to boost the reaction kinetics. In Figure S7a, the full XPS spectrum shows that ZnO-QDs-VN contains V, N, O, and Zn elements, while VN contains only N and V. The quantification of their contents is shown in Tables S1 and S2; the content of Zn in ZnO-QDs-VN is about 1.3 at.%. In Zn 2p spectra of ZnO-QDs-VN (Figure S7b), the peaks at 1021.9 and 1045.1 eV belong to Zn 2p_3/2_ and Zn 2p_1/2_ of ZnO, respectively [23], while there are no obvious peaks in the Zn 2p spectra of VN, indicating that ZnO-QDs were completely removed by acid etching. In V 2p spectra (Figure 2e), ZnO-QDs-VN displays three deconvoluted peaks, attributed to V^2+^ (514.2 eV), V^3+^ (516.1 eV), and V^4+^ (517.4 eV) [24]. It is noteworthy that the binding energy (BE) of V in ZnO-QDs-VN is higher than that of VN, indicating that the charge is transferred from VN to ZnO-QDs. These findings align with the results of M–S plots [25]. Moreover, the N1s (Figure 2f) further support the views, where one can find that the BE of N1s in ZnO-QDs-VN is the positive shift of 0.2 eV compared to VN. Moreover, the differential charge density analysis (Figure 2g and Figure S8) demonstrates that the electron cloud density in VN is diminished following the formation of a heterojunction, whereas the electron cloud density in ZnO is augmented. The electron-rich region has the potential to enhance the affinity for Li, while the electron-deficient region may improve the affinity for S [26,27]. The dual effects in ZnO-QDs-VN can boost the conversion of LiPSs in LSBs [21].

### 3.2. Overview of Calculation Details and Methods

Density functional theory (DFT) calculations were performed in the CASTEP code of Material Studio 2019. The generalized gradient approximation (GGA) and the Perdew–Burke–Ernzerhof (PBE) function were used for exchange-correlation energy. The k-point mesh and cutoff energy for plane-wave expansions were set to “3 × 2 × 1” and 450 eV, respectively. The atomic positions were relaxed until the force on each atom was under 0.03 eV Å^−1^, and the convergence tolerance of the energy was set to 10^−5^ eV. To avoid interplanar interactions, a vacuum space of 15 Å was applied to each slab. VN (200) and ZnO (101) were used to construct the heterostructure of ZnO-QDs-VN. All the heterostructures were matched under a 5% matching error.

### 3.3. Spin Configuration−Orbital Orientation

To theoretically investigate the influence of ZnO on the electron alignment within V3d-orbitals, we analyzed the electron spin states through projected density of states (PDOS) calculations. By integrating the PDOS curve near this critical energy point, one can ascertain the occupancy numbers for these orbitals, thereby elucidating changes in electron distribution among different orbital types [5]. As illustrated in Figure 2h,i, the PDOS analysis indicated that, compared to the pure VN model, there was a significant reduction in electron occupancy within both low-energy t_2g_ and high-energy e_g_ orbitals in the ZnO-QDs-VN model. This reduction in electron count primarily stems from ZnO’s impact on V 3d-orbitals, particularly due to bonding interactions between oxygen atoms and vanadium atoms within ZnO. The V 3d-orbital undergoes splitting into lower-energy t_2g_ and higher-energy e_g_ levels as a result of crystal field effects. In isolation, under relatively weak crystal fields present in VN models, energy separation between t_2g_ and e_g_ levels remains minimal, resulting in a relatively stable electron distribution. However, upon integration with ZnO-QDs, oxygen atoms significantly alter 3d-orbital energy distributions by interacting with V 3d electrons, a process attributed to variations stemming from oxygen atom electronegativity coupled with ZnO crystal field influences, which intensify splitting among V 3d levels while forming more discrete d-orbital groups. Consequently, under the enhanced induction of the crystal field effect with the participation of ZnO, electrons transition from a high-energy configuration (e_g_) to a low-energy configuration after splitting, and the orbital occupancy of the high-energy e_g_ is significantly reduced. Furthermore, alongside diminished occupation rates within high-energy e_g_ levels lies a concurrent decline observed within low-energy t_2g_ orbitals. In the ZnO-QDs-VN model, the splitting of the V 3d-orbitals encompasses not only the energy elevation of the e_g_ orbit but also alterations in the energy levels of the t_2g_ orbitals. Consequently, some electrons may transition from their original t_2g_ orbital to other, lower-energy orbitals, thereby diminishing electron occupancy within the t_2g_ orbital. PDOS calculations revealed that within this model framework (ZnO-QDs-VN), due to electron transfer from higher-energy to lower-energy orbitals, there is also a decrease in low-energy orbital occupancy, which indicates an ongoing rearrangement of electron spins as well.

To further explore structural active sites within ZnO-QDs-VN, we concentrated on coupling dynamics between the 3d orbital of the V element and key intermediate orbitals throughout the reaction processes. As shown in Figure 2j,k, vanadium possesses five d-orbitals: d_x²−y²_, d_z²_, d_xy_, d_xz_, and d_yz_, all exhibiting distinct energy distributions influenced by ligand field effects within an ideal octahedral coordination environment provided by ZnO-QDs-VN [28]. During the operation of lithium-sulfur batteries, lithium polysulfides (LiPSs) and Li_2_S are two important reaction intermediates. The electronic structure of LiPSs is mainly determined by the 3p orbitals of sulfur atoms [29]. According to the principle of orbital symmetry, the 3d_z2_-orbital of Voh^2+^ undergoes significant d-p hybridization due to its strong symmetry match with the p_z_ orbital of S, forming σ bonds and σ* bonds. At the same time, the 3d_xz_ and 3d_yz_ orbitals of Voh^2+^ are hybridized with the 3p_x_ and 3p_y_ orbitals of S, forming weaker π and π* bonds. Compared with σ bonds, π bonds have a lower energy to form, but they play a key role in regulating the stability of the intermediate and the advancement of the reaction process [30]. It is this d-p hybridization phenomenon that determines the specific behavior of the ZnO-QDs-VN structure in the catalytic reaction through the bonding strength of different orbitals. Based on ligand field theory and molecular orbital theory, within this framework, it is observed that intermediate spin states in the ZnO-QDs-VN structure (2.426) exhibit greater stability compared to those in the VN model (2.306). This phenomenon indicates that ZnO-QDs-VN can capture LiPSs more effectively and significantly inhibit the LiPS shuttle effect.

### 3.4. Absorption and Catalytic Conversion Mechanism

**Figure 3:**
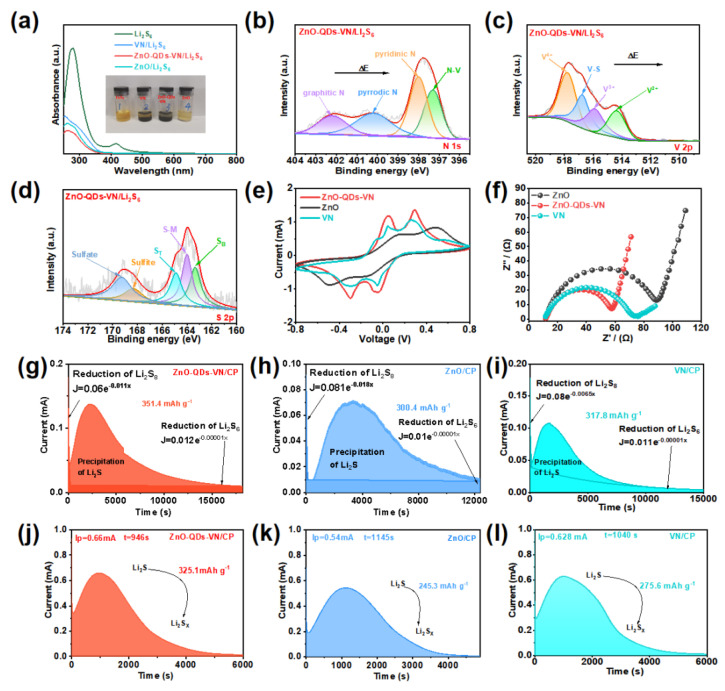
(**a**) UV absorption spectra of Li_2_S_6_ solutions assimilated by VN, ZnO-QDs-VN, and ZnO powders, in which the illustration is a direct result of Li_2_S_6_ adsorption experiments. High-resolution XPS profiles of (**b**) N 1s, (**c**) V 2p, and (**d**) S 2p regions of ZnO-QDs-VN after soaking in Li_2_S_6_. (**e**) CV of symmetric cells for ZnO-QDs-VN, VN, and ZnO. (**f**) Nyquist plots of ZnO-QDs-VN, VN, and ZnO. Chronoamperometric profiles of nucleation measurements of (**g**) ZnO-QDs-VN, (**h**) ZnO, and (**i**) VN. Chronoamperometric profiles of Li_2_S dissolution tests for (**j**) ZnO-QDs-VN, (**k**) ZnO, and (**l**) VN.

The LiPS adsorption capacity of ZnO-QDs-VN, VN, and ZnO was analyzed by static adsorption experiments in Li_2_S_6_ solution (3 × 10^−3^ M). The Figure 3a inset shows that the ZnO-QDs-VN/Li_2_S_6_ solution becomes nearly transparent after 3 h. Nevertheless, the VN/Li_2_S_6_ solution still shows a deep yellow color at the same time. The color of the ZnO/Li_2_S_6_ solution was between that of the ZnO-QDs-VN/Li_2_S_6_ solution and VN/Li_2_S_6_ solution. The ultraviolet (UV) spectra (Figure 3a) confirmed the results, where the ZnO-QDs-VN exhibited a much weaker absorption peak at around 370 nm compared to VN and ZnO. The results further proved that the interaction between ZnO-QDs and VN can greatly enhance the adsorption capacity towards LiPSs. To gain insight into the reasons for the increased adsorption capacity, XPS tests were performed after the adsorption experiments. In the full XPS spectra (Figure S9a), a new formation peak (Li 1s) was evident, indicating that LiPSs are adsorbed on the sample. In Figure S9b, the BE of the peaks of Zn 2p_1/2_ and Zn 2p_3/2_ in ZnO-QDs-VN/Li_2_S_6_ are negatively shifted compared with ZnO-QDs-VN. In addition, the BE of both N 1s and V 2p_3/2_ is also shifted. Interestingly, compared to ZnO-QDS-VN, a new V-S peak appeared at 516.8 eV in V 2p_3/2_ of ZnO-QDS-VN/Li_2_S_6_ [30,31] (Figure 3b,c). The results suggest that there is a strong chemisorption between Li_2_S_6_ and ZnO-QDs-VN. As shown in Figure 3d, the S 2p spectrum exhibits two peaks at 163.4 and 164.6 eV, corresponding to the “bridging” sulfur (S_B_) and the “terminal” sulfur (S_T_), respectively [32]. Meanwhile, the peak located at 164 eV was identified as the formation of the sulfur–metal (S–M) bond, further confirming the chemical interaction between ZnO-QDs-VN and LiPSs. Moreover, due to the surface redox reaction between ZnO-QDs-VN and LiPSs, additional peaks were observed at 168.32 and 169.72 eV, which are attributed to the thiosulfate and sulfate, suggesting the good catalytic activity of ZnO-QDs-VN [33].

Then, the catalytic performance of various materials was analyzed through a symmetric cell test. In the cyclic voltammetry (CV) curve (Figure 3e), the ZnO-QDs-VN symmetric cell showed two pairs of distinct redox peaks, which belong to a multi-step conversion of LiPSs. A comparable phenomenon was observed in both the symmetric cells of VN and ZnO, but the redox current peak was lower than that of ZnO-QDs-VN, and the corresponding potential revealed hysteresis, indicating that the heterojunction can significantly improve the redox reaction kinetics of LiPSs [34]. The electrochemical impedance curve (EIS) was further calculated to analyze the performance, where the high-frequency region is typically associated with charge transfer resistance (R_ct_), while the low-frequency region is employed to quantify the diffusion resistance (Z_w_) of lithium ions [35,36]. In Figure 3f, one can find that the ZnO-QDs-VN symmetric cells exhibit lower charge transfer resistance and greater diffusion resistance among all samples, which reveals that forming heterojunctions can also improve the charge/Li^+^ transfer.

In addition, we also perform Li_2_S nucleation experiments to further analyze the catalytic activity of different samples towards the conversion of LiPSs. Figure 3g–i illustrate that the ZnO-QDs-VN electrode exhibits a higher precipitation peak (Ip = 0.14 mA) and a faster nucleation time (t = 2323 s) in comparison to ZnO (Ip = 0.071 mA, t = 3357 s) and VN (Ip = 0.1074 mA, t = 2830 s). Moreover, the Li_2_S nucleation ability of the ZnO-QDs-VN electrode (351.4 mAh g^−1^) has a significantly higher Li_2_S nucleation capacity than that of ZnO (300.4 mAh g^−1^) and VN (317.8 mAh g^−1^), confirming that the presence of ZnO-QDs enhances the kinetics and catalytic activity of LiPS conversion to Li_2_S. Subsequently, the Li_2_S dissolution experiment was conducted, as illustrated in Figure 3j–l. Compared with the electrode of ZnO (Ip = 0.54 mA, 245.3 mAh g^−1^, t = 1145 s) and VN (Ip = 0.628 mA, 275.6 mAh g^−1^, t = 1040 s), the ZnO-QDs-VN electrode exhibited a greater dissolution current response (Ip = 0.66 mA), a higher dissolution capacity (325.1 mAh g^−1^), and an earlier dissolution time (946 s). The above results demonstrate that ZnO-QDs-VN possesses superior redox kinetics for bidirectional sulfur conversion.

### 3.5. Electrochemical Performance of ZnO-QDs-VN Heterostructure Composite Materials

**Figure 4:**
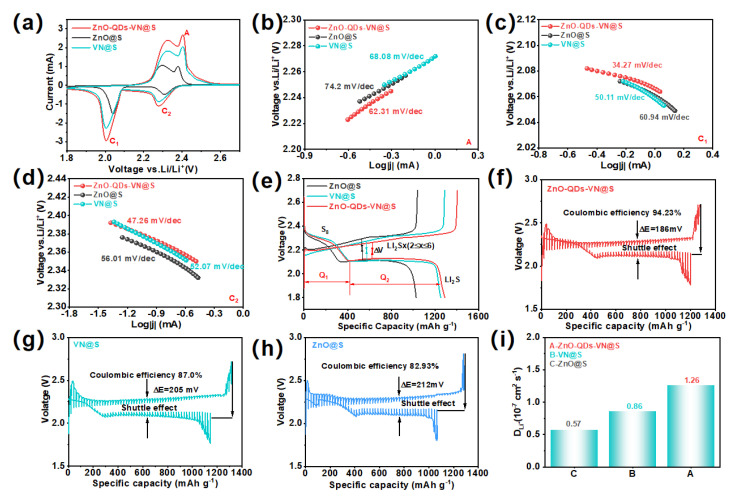
(**a**) The CV curves of the ZnO-QDs-VN@S, VN@S, and ZnO@S at the rate of 0.1 mV s^−1^ in the voltage range of 1.7–2.8 V. Tafel plots of oxidation peak at around (**b**) A, reduction peak at around (**c**) C_1_, and (**d**) C_2_ of ZnO-QDs-VN@S, ZnO@S, and VN@S. (**e**) Typical charge and discharge platform for ZnO-QDs-VN@S, VN@S, and ZnO@S at 0.1 C. GITT voltage curve of (**f**) ZnO-QDs-VN@S, (**g**) VN@S, and (**h**) ZnO@S cathode. (**i**) Lithium ion diffusion coefficients calculated from GITT tests for different cathodes.

The electrochemical properties of the as-prepared samples were further characterized by assembling the ZnO-QDs-VN@S cathode (loading of 1.2 mg cm^−2^). Figure 4a depicts the CV profiles of ZnO-QDs-VN@S, ZnO@S, and VN@S cathodes under the voltage window of 1.7–2.8 V with a scan rate of 0.1 mV s^−1^. Two distinct reduction peaks were observed in the CV curve at 2.0 and 2.28 V, which are attributed to the reduction conversion of S_8_ to higher-order LiPSs, subsequently undergoing a conversion to Li_2_S_x_ (4 ≤ X ≤ 8), and finally to Li_2_S_2_/Li_2_S. Correspondingly, there were two distinct oxidation peaks at 2.32 and 2.4 V, attributed to the conversion of Li_2_S_2_/Li_2_S to Li_2_S_x_ [13]. The ZnO-QDs-VN@S exhibited higher current density and lower reaction polarization during the sulfur redox processes, further indicating the superior redox kinetics of the ZnO-QDs-VN@S cathode. Then, linear voltammetry (LSV) was used to investigate the catalytic performance for the bidirectional sulfur conversion of LiPSs (Figure S10a–c), in which the ZnO-QDs-VN@S cathode had a higher current than those of VN@S and ZnO@S in the potential range of 2.20–2.32 V, 2.02–2.10 V, and 2.28–2.44 V. The corresponding Tafel plots based on CV curves (Figure 4b–d) showed that the Tafel slope of the ZnO-QDs-VN@S cathode at A, C_1_, and C_2_ peaks were 62.31, 34.27, and 47.26 mV dec^−1^, respectively, lower than those of ZnO@S (74.2, 60.94, and 56.01 mV dec^−1^) and VN@S (68.08, 50.11, and 52.07 mV dec^−1^), further indicating that the ZnO-QDs-VN@S can significantly enhance the kinetics of bidirectional sulfur reactions.

To further evaluate the diffusive ability of Li^+^ during the reaction, the CV test was performed with scanning rates from 0.1 to 0.5 mV s^−1^ (Figure S11a–c). As the scanning rate was increased, the oxidation peaks were observed to shift to higher potentials, while the reduced peaks shifted to lower potentials. The shift values of ZnO-QDs-VN@S at the A, C_1_, and C_2_ peaks were 41, 79, and 17 mV, smaller than those of ZnO@S (63, 82, 40 mV) and VN@S (60, 81, 26 mV), which is a notable indicator of enhanced Li^+^ diffusivity and reaction kinetics. In Figure S12a–c, it can be found that the oxidation/reduction peaks’ current densities are linearly related to the square root of the scanning speed. The Li^+^ diffusion coefficient (D_liq_) was estimated based on the classical Randles–Sevcik equation [37] (Equation (S1)). As shown in Figure S13, the peaks of ZnO-QDs-VN@S at A, C1, and C2 were 5.56 × 10^−7^, 1.73 × 10^−7^, and 0.9 × 10^−7^, respectively, higher than those of ZnO@S (A: 2.84 × 10^−7^, C1: 1.25 × 10^−7^, C2: 0.63 × 10^−7^) and VN@S (A: 4.8 × 10^−7^, C1: 1.39 × 10^−7^, C2: 0.85 × 10^−7^).These results indicate that ZnO-QDs-VN@S demonstrates higher lithium ion mass transfer efficiency in the electrolyte and superior interfacial ion transport kinetics. Meanwhile, they also reflect that ZnO-QDs-VN@S is favorable for enhancing Li^+^ diffusion capability.

Figure S14 depicts the three-times CV curves of the ZnO-QDs-VN@S cathode, in which each curve overlaps very well, suggesting that the ZnO-QDs-VN@S cathode effectively alleviates the dissolution of LiPSs and improves the reversible conversion of the charge–discharge process. In constant-current charge–discharge (GCD) curves of as-prepared samples (Figure 4e), there were two platforms in the discharge curve; the first platform was the conversion of S_8_ to Li_2_S_x_ (4 ≤ X ≤ 8), and the second platform was the conversion of long-chain LiPSs to short-chain Li_2_S_2_/Li_2_S [38,39]. The discharge capacities of ZnO-QDs-VN@S in the first and second platforms were 398.6 and 891.2 mAh g^−1^, much higher than those of ZnO@S (335.9, 691.5 mAh g^−1^) and VN@S (392.7, 837.2 mAh g^−1^). The catalytic efficiency of LiPSs can be further analyzed by the emission platform capacity ratio between the first and second platforms [40], and the corresponding platform capacity ratios were 2.24, 2.05, and 2.13 for ZnO-QDs-VN@S, ZnO@S, and VN@S, respectively (Table S3). In addition, the ZnO-QDs-VN@S cathode displayed a smaller polarization potential of only 132 mV, superior to the ZnO@S (ΔV = 169 mV) and VN@S (ΔV = 151 mV) cathodes. The results further prove that ZnO-QDs-VN@S possesses excellent catalytic ability for polysulfide redox reactions.

**Figure 5:**
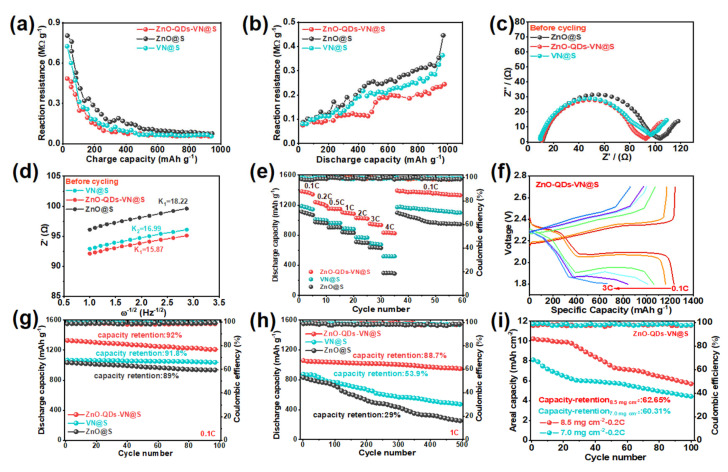
Reaction resistance of the LSBs with various separators during the (**a**) charge and (**b**) discharge processes. (**c**) EIS curves of ZnO-QDs-VN@S, ZnO@S, and VN@S (the data are the means of three independent experiments ± 2.8 Ω). (**d**) The relationship between Z’ and ω^1/2^ with a frequency range between 3.5 and 0.5 Hz (symbols, real data; lines, fitting curves) of ZnO-QDs-VN@S, ZnO@S, and VN@S cathodes. (**e**) Charge/discharge profiles of LSBs assembled with ZnO-QDs-VN samples at 0.1–4.0 current densities. (**f**) Charge/discharge curves of ZnO-QDs-VN@S, VN@S, and ZnO@S in different current densities (The red, orange, green, cyan, purple, and blue lines represent the charge-discharge curves of the ZnO-QDs-VN@S cathode at 0.1 C, 0.2 C, 0.5 C, 1.0 C, 2.0 C, and 3.0 C, respectively). (**g**) Cycle performance of ZnO-QDs-VN@S, VN@S, and ZnO@S cathode at 0.1 C. (**h**) Cycle performance of ZnO-QDs-VN@S at 1.0 C. (**i**) The areal capacity of ZnO-QDs-VN@S cathodes under 0.2 C with high sulfur loading.

The internal resistance of the ZnO-QDs-VN@S cathode during the discharge and charge cycles was measured utilizing the constant current intermittent titration technique (GITT) [41]. In Figure 4f–h, the ZnO-QDs-VN@S cathode exhibits two discharge platforms with a higher discharge capacity of 1200.1 mAh g^−1^, smaller polarization potential ΔE = 186 mV, and better Coulomb efficiency of 94.23% compared to the VN@S cathode (1144.2 mAh g^−1^, 87.0%, 205 mV) and ZnO@S cathode (1069 mAh g^−1^, 82.93%, 212 mV), indicating the high sulfur utilization in the ZnO-QDs-VN@S cathode. According to the results of the conducted GITT tests, the calculation formula for the lithium ion diffusion coefficient (D_Li_^+^) was calculated with the Wagner equation [40] (Equation (S2)). As shown in Figure 4i, the Li^+^ diffusion coefficient of ZnO-QDs-VN@S was 1.26 × 10^−7^, which was higher than that of ZnO@S (0.57 × 10^−7^) and VN@S (0.86 × 10^−7^), respectively. It reflects the real kinetic characteristics of the ZnO-QDs-VN@S electrode under limited diffusion conditions, contributing to the enhancement of the battery’s cycle stability. Furthermore, the internal resistance during lithiation/dehydrogenation was measured using the following equation [22,42] (Equation (S3)). As illustrated in Figure 5a,b, the ZnO-QDs-VN@S cathode exhibited a reduction in resistance during the charging and discharging processes compared with the VN@S and ZnO@S cathodes, further suggesting that the ZnO-QDs-VN@S cathode is capable of enhancing the chemical anchoring and catalytic reaction of LiPSs.

Additionally, EIS tests were conducted before and after 100 and 150 cycles of the battery to assess its conductivity. In the Nyquist plots (Figure 5c,d), the ZnO-ODs-VN@S cathode exhibits the lowest Ret value among all samples. This finding demonstrates that the ZnO-ODs-VN battery possesses excellent fast charge and ion transfer capabilities, accelerating the redox reaction kinetics of sulfur. Due to the penetration of the electrolyte, the active material on the electrode surface was redispersed after cycling. Therefore, both R_ct_ and Z_w_ were smaller than those before cycling (Figure S15a,b) [11]. By comparing the R_ct_ and Z_w_ of the ZnO-QDs-VN@S, VN@S, and ZnO@S cathodes before and after cycling, the study shows that with the increase in battery cycle times, the charge transfer resistance (Rct) presents a downward trend. This phenomenon may be related to the following mechanisms. First, during the cycling process, more stable catalytic sites gradually form at the interface between the active material and the carrier (ZnO-QDS-VN@S structure), accelerating the electrochemical reaction kinetics and thereby reducing the Rct. Second, although the cycle may be accompanied by some structural changes, the unique porous structure in the material design (the highly conductive skeleton of VN) continuously optimizes the lithium-ion/electron transport path, offsetting part of the increase in mass transfer resistance. Additionally, the results further confirm that the ZnO-QDs-VN@S cathode exhibits a higher electron transfer rate and stronger electron transfer capability.

Figure 5e shows the rate performance of the prepared materials. The ZnO-QDs-VN@S cathode expressed high specific capacities at 0.1, 0.2, 0.5, 1.0, 2.0, 3.0, and 4.0 C, which were 1382.9, 1189.9, 1148.7, 1084.8, 1019.2, 935.3, and 800.5 mAh g^−1^, respectively, higher than those of the ZnO@S cathode and VN@S cathode. Upon returning the current rate to 0.1 C, the specific capacity could be recovered to 1330.1 mAh g^−1^, representing 96.23% of the initial capacity, demonstrating exceptional rate performance. The corresponding charge–discharge curves at different current densities (Figure 5f and Figure S16a,b) showed that the ZnO-QDs-VN@S cathode still expressed two well-defined discharge voltage plateaus even at the ultra-high 3.0 C, which further indicates the effective adsorption and conversion of LiPSs on the ZnO-QDs-VN surface. Moreover, by comparing the differences in the corresponding GCD spectra of the prepared samples (Figure S16c), ZnO-QDs-VN@S showed a lower polarization value at different current densities.

Figure 5g illustrates the long-term cycling durability tests, which demonstrated that the initial capacity of the ZnO-QDs-VN@S cathode was 1329.3 mAh g^−1^ at 0.1 C. Following 100 cycles, the specific capacity was observed to remain at 1210.1 mAh g^−1^, while the coulombic efficiency was recorded at 92%. These values were better than those of the ZnO@S cathode (1034.4 mAh g^−1^, 89%) and VN@S cathode (1038.7 mAh g^−1^, 91.8%). At 0.5 C, the ZnO-QDs-VN@S cathode also displayed a high initial specific capacity of 1135.6 mAh g^−1^, and could still maintain a capacity of 911.89 mAh g^−1^ after 500 cycles with a coulombic efficiency of 84.3% (Figure S17a). Moreover, in Figure 5h, one can find that the ZnO-QDs-VN@S cathode also exhibits excellent stability even at 1.0 C, which can be maintained at 984.4 mAh g^−1^ after 500 cycles with a high coulombic efficiency of 88.7%. In contrast, the capacity of the ZnO@S and VN@S cathodes underwent a sharp decrease after 500 cycles, dropping to only 259.6 and 471.4 mAh g^−1^, respectively. Even at 2.0 and 3.0 C (Figure S17b,c), the ZnO-QDs-VN@S cathode still exhibited remarkable cycling stability, with specific capacities remaining at 840.5 (0.044% decay per cycle) and 687.9 mAh g^−1^ (0.056% decay per cycle) after 500 cycles, respectively.

The performance of the ZnO-QDs-VN@S cathode under high sulfur mass loading was further investigated. In Figure 5i, ZnO-QDs-VN@S with high S loading (7.0 mg cm^−2^) exhibits a high initial areal capacity of 8.107 mAh cm^−2^ at 0.2 C. Moreover, when the S loading mass further increases to 8.5 mg cm^−2^, ZnO-QDs-VN@S also provides a relatively high initial area capacity of 10.258 mAh cm^−2^, and after 100 cycles, the capacity retention rate is 62.65%. However, the VN@S cathode under high S loading displays a low initial area capacity of only 6.042 and 7.308 mAh cm^−2^, with capacity retention rates of 54.05% and 52.25%, respectively (Figure S18). The findings demonstrate that ZnO-QDs-VN, which exhibits high performance as a sulfur host, is capable of facilitating the sulfur reaction kinetics even under high sulfur loading. Its capability even surpasses that of the majority of advanced sulfur host materials that have been reported in LSBs (Table S4).

## 4. Conclusions

In short, we have successfully synthesized an in situ nanoreactor of a zinc oxide dots-modified vanadium nitride flower-like heterojunction (Zn-QDs-VN) with a phase separation strategy as a multifunctional sulfur host material for LSBs. Various characterization and electrochemical tests indicated that the unique heterostructure of ZnO-QDs-VN increases the sulfur loading, promotes the bidirectional catalytic conversion of LiPSs, effectively suppresses the shuttle effect of LiPSs, enhances the rapid transfer of electrons/ions, and accelerates the entire redox reaction kinetics. Further theoretical computations demonstrated that ZnO-QDs-VN with optimized intermediate spin states can form a stable LiS* bond sequence, which can significantly promote the adsorption and conversion of LiPSs and reduce the energy barrier in the battery reaction. As a consequence, the ZnO-QDs-VN@S cathode exhibits excellent electrochemical performance. The ZnO-QDs-VN@S cathode can reach a high initial specific capacity of 960.8 mAh g^−1^ even at 3.0 C and maintains a specific capacity of 687.9 mAh g^−1^ after 500 cycles, with a decay rate of 0.056% per cycle. Moreover, under high sulfur loading (8.5 mg cm^−2^), the ZnO-QDs-VN@S cathode can maintain a high initial area capacity of 10.258 mAh cm^−2^ at 0.2 C and has good durability (CR of 62.65% after 100 cycles). The study provides a new method for designing heterojunctions to achieve high-performance LSBs.

## 5. Prospects

In summary, due to poor cycle stability and the obvious “shuttle effect”, the commercialization process for LSBs has encountered severe challenges. To address this issue, this study focused on constructing a ZnO-QDs-VN heterostructure, aiming to accelerate the catalytic conversion rate of LiPSs and deeply analyze the mechanism of the ZnO-QDs-VN catalyst on LiPSs. Meanwhile, the internal structure of the ZnO-QDs-VN heterojunction and its intrinsic relationship with the adsorption/catalytic behavior of LiPSs were also deeply studied. Although certain research achievements have been made, it is still necessary to conduct in-depth and systematic research on the related mechanisms. Currently, the following prospects are proposed for subsequent research:

1. Deepen the analysis of application potential. Through systematic research on industry data (such as production costs and process parameters of similar materials), combined with existing experimental data, conduct a preliminary feasibility assessment of the synthetic method from the perspectives of raw material economy and process complexity, and clarify the gap between current research and practical application.
2. Strengthen cross-disciplinary cooperation. We have reached initial cooperation agreements with certain enterprises and plan to jointly conduct pilot-scale amplification experiments in subsequent research, optimize the synthesis process in line with the demands of the enterprises, and verify the performance of the materials under actual working conditions.
3. Adjust the research focus. In line with recommendations, we will reduce some repetitive electrochemical characterization content and instead focus on the synthesis mechanism during the synthesis process, providing theoretical support for subsequent process optimization through mechanism analysis.

## Data Availability

The original contributions presented in the study are included in the article/supplementary material, further inquiries can be directed to the corresponding author.

## Supplementary Materials

The following supporting information can be downloaded at: https://www.mdpi.com/article/10.3390/ma18112639/s1, Figure S1: SEM images for (a) VN, and (b) ZnO-QDs-VN@S, Figure S2: XRD patterns of (a) ZnO-QDs-VN, VN, and ZnO, (b) ZnO-QDs-VN@S, Figure S3: TEM images of ZnO-QDs-VN at different magnifications, Figure S4: The XRD patterns of the refined (a) VN, (b) ZnO-QDs-VN, and (c) ZnO, Figure S5: BET adsorption-desorption isotherms of (a) ZnO-QDs-VN, (b) VN, (c) ZnO-QDs-VN@S, Figure S6:TGA of ZnO-QDs-VN@S, VN@S, and ZnO@S. Figure S7: (a) XPS survey scan of ZnO-QDs-VN, (b) High-resolution XPS spectra of Zn 2p, Figure S8: Schematic diagram of differential charge density of (a) ZnO, (b) ZnO-QDs-VN, Figure S9: (a) XPS survey scan of ZnO-QDs-VN/Li_2_S_6_, High-resolution XPS profiles (b) Zn 2p regions of ZnO-QDs-VN after soaking in Li_2_S_6_, Figure S10: LSV curves at around (a) A, (b) C_1_, and (c) C_2_ of ZnO-QDs-VN@S, ZnO@S, and VN@S, Figure S11: CV curves of ZnO-QDs-VN@S, ZnO@S, and VN@S at the rate of 0.1–0.5 mV s^−1^, Figure S12: (a) A, (b) C_1_, and (c) C_2_ peak current and square root of scan rate, Figure S13: Li^+^ diffusion coefficients calculated from CV curves for different cathodes, Figure S14: Three cycles of CV curves of ZnO-QDs-VN@S cathode at 0.5 mV s^−1^, Figure S15: (a) EIS curves of ZnO-QDs-VN@S, ZnO@S, and VN@S after 100 cycles (data are mean ± 2.2 Ω from three independent experiments), (b) EIS curves of ZnO-QDs-VN@S, ZnO@S, and VN@S after 150 cycles (data are mean ± 1.8 Ω from three independent experiments), Figure S16: (a,b) Charging and discharging curves of LSBs assembled at different speeds with ZnO@S and VN@S cathodes, (c) The voltage differences of LSBs fabricated with distinct cathode materials at different rates, Figure S17: (a–c) Cycle performance of ZnO-QDs-VN@S at 0.5 C, 2.0 C, and 3.0 C, Figure S18: The areal capacity of VN@S cathodes under 0.2 C with high sulfur loading, Table S1: High-resolution fitting parameter table of ZnO-QDs-VN. Table S2: High-resolution fitting parameter table of VN, Table S3: The discharge capacity and ratio between the two discharge platforms, Table S4: Comparison of the ZnO-QDs-VN@S performance with other published Works. Equation (S1): Calculation of D_Li_^+^ from CV curves, Equation (S2): Calculation of D_Li_^+^ from GITT curves, Equation (S3): Calculation of internal resistance from GITT. Refs. [43,44,45,46] are cited in Supplementary Materials.
