# Supplementary material for: In Situ Phase Separation Strategy to Construct Zinc Oxide Dots-Modified Vanadium Nitride Flower-like Heterojunctions as an Efficient Sulfur Nanoreactor for Lithium-Sulfur Batteries
Source: Materials (Basel). 2025 Jun 4;18(11):2639. doi: 10.3390/ma18112639 (PMC12156793; doi:10.3390/ma18112639)
Supplement: Supplementary file 1 [file materials-18-02639-s001.zip › materials-3592074-supplementary.pdf]

Supplementary Materials for

In Situ Phase Separation Strategy to Construct Zinc Oxide Dots-modified Vanadium Nitride Flower-like Heterojunctions as an Efficient Sulfur Nanoreactor for Lithium-Sulfur Batteries

Ningning Chen [1], Wei Zhou [1], Minzhe Chen [1], Ke Yuan [1], Haofeng Zuo [1], Aocheng Wang [1], Dengke Zhao [2,], Nan Wang [3], and Ligui Li [1,*]*

[1] New Energy Research Institute, College of Environment and Energy, South China University of Technology, Guangzhou 510006, China.

[2] School of Materials Science and Engineering, Henan Normal University, Xinxiang 453007, China.

[3] Siyuan laboratory, Guangzhou Key Laboratory of Vacuum Coating Technologies and New Energy Materials, Guangdong Provincial Engineering Technology Research Center of Vacuum Coating Technologies and New Energy Materials, Guangdong Provincial Key Laboratory of Nanophotonic Manipulation, Department of Physics, Jinan University, Guangzhou, Guangdong 510632, China.

*The Corresponding authors: zhaodengke@htu.edu.cn (D. K. Zhao); esguili@scut.edu.cn (L.G. Li).

The PDF file includes:

Materials and Methods

Figs. S1 to S18

Tables S1 to S4

Equations S1 to S3

Supplementary Figures

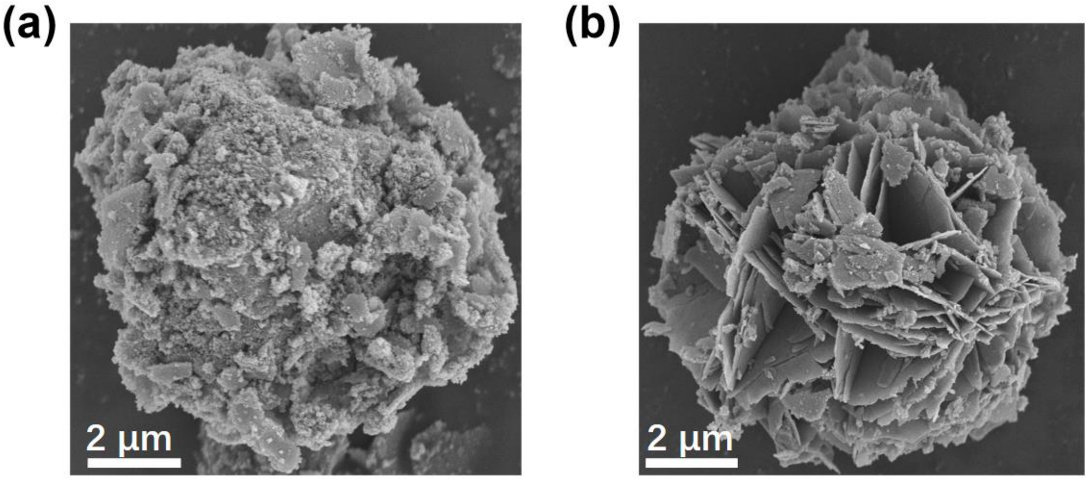

Figure S1. SEM images for (a) VN, and (b) ZnO-QDs-VN@S.

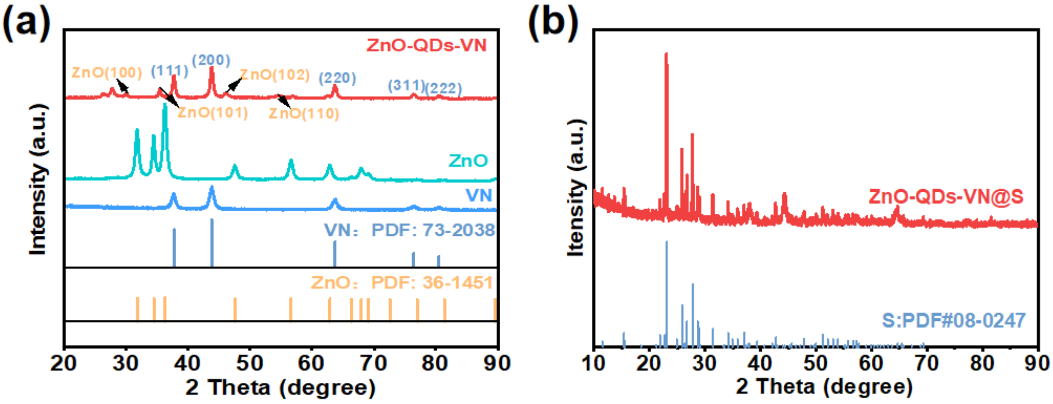

Figure S2. XRD patterns of (a) ZnO-QDs-VN, VN, and ZnO. (b) ZnO-QDs-VN@S.

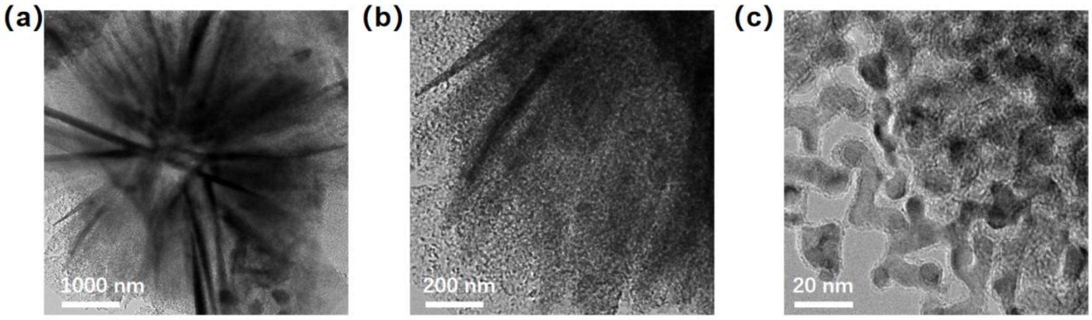

Figure S3. TEM images of ZnO-QDs-VN at different magnifications.

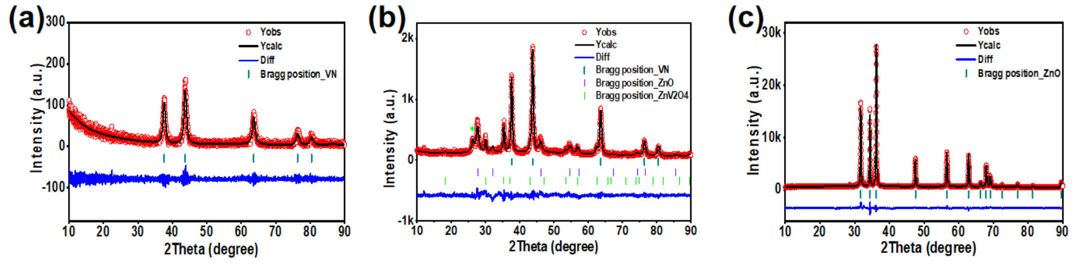

Figure S4. The XRD patterns of the refined (a)VN, (b) ZnO-QDs-VN, and (c) ZnO.

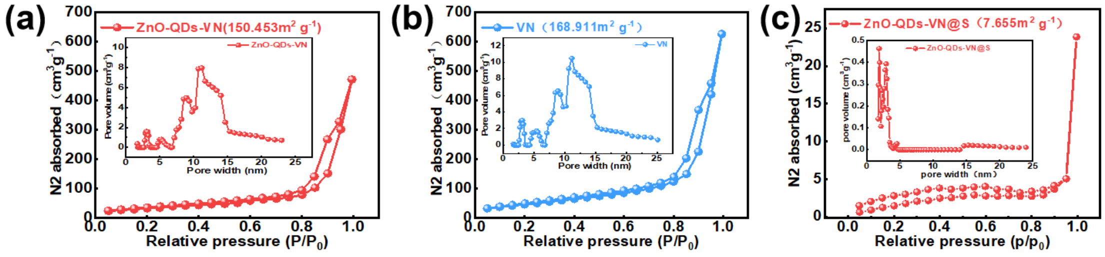

Figure S5. BET adsorption-desorption isotherms of (a) ZnO-QDs-VN, (b) VN, (c) ZnO-QDs-VN@S.

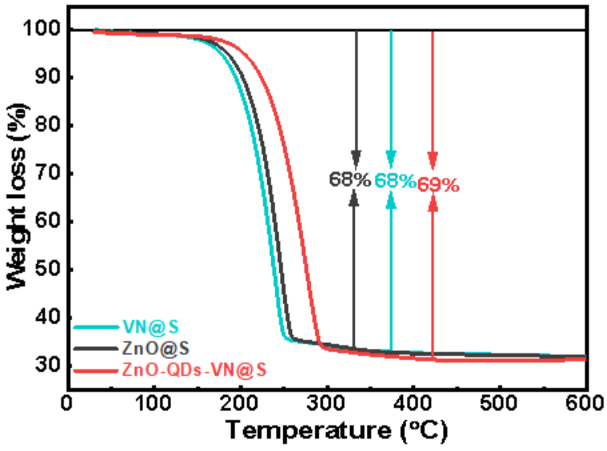

Figure S6.TGA of ZnO-QDs-VN@S, VN@S, and ZnO@S.

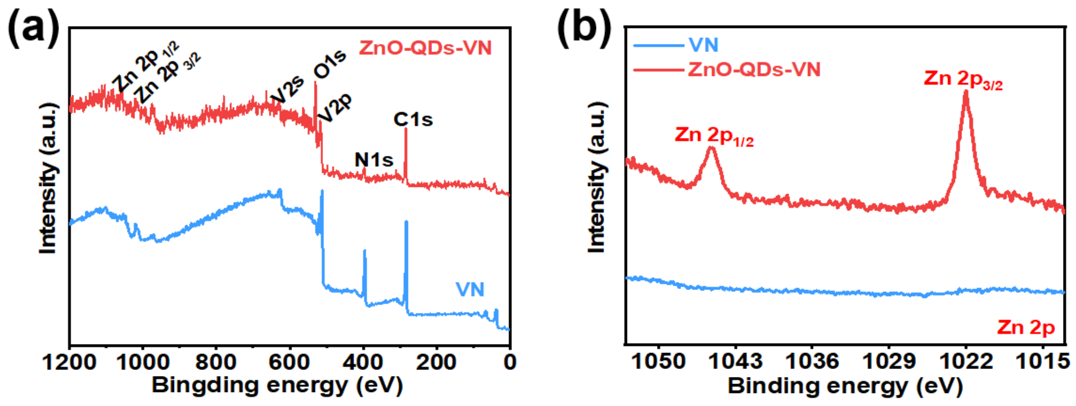

Figure S7. (a) XPS survey scan of ZnO-QDs-VN. (b) High-resolution XPS spectra of Zn 2p.

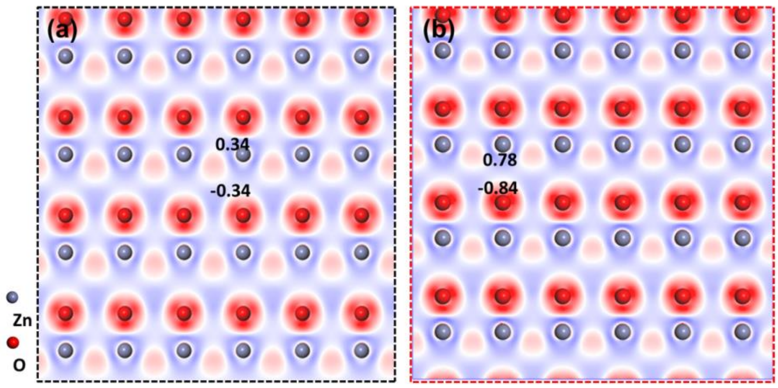

Figure S8. Schematic diagram of differential charge density of (a) ZnO, (b) ZnO-QDs-VN.

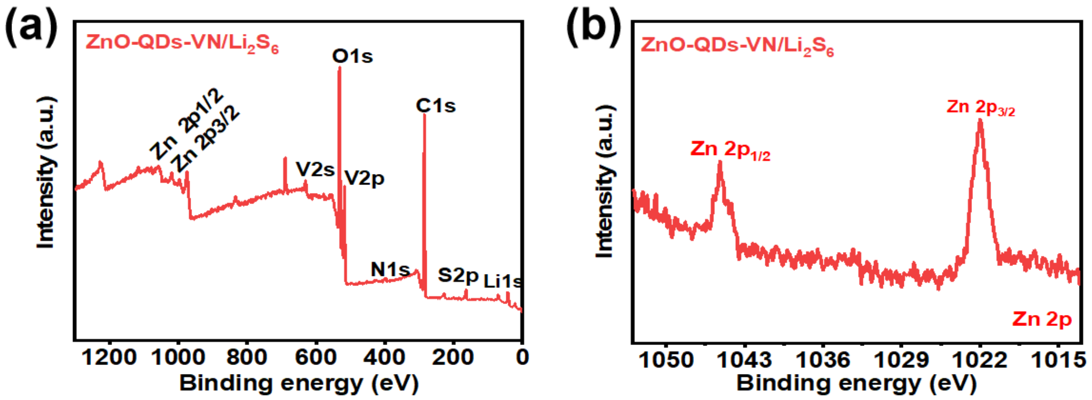

Figure S9. (a) XPS survey scan of ZnO-QDs-VN/Li_2S_6. High-resolution XPS profiles (b) Zn 2p regions of ZnO-QDs-VN after soaking in Li_2S_6.

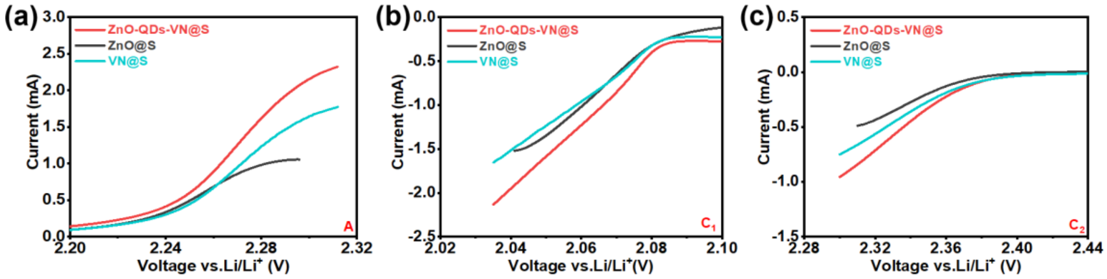

Figure S10. LSV curves at around (a) A, (b) C_1, and (c) C_2 of ZnO-QDs-VN@S, ZnO@S, and VN@S.

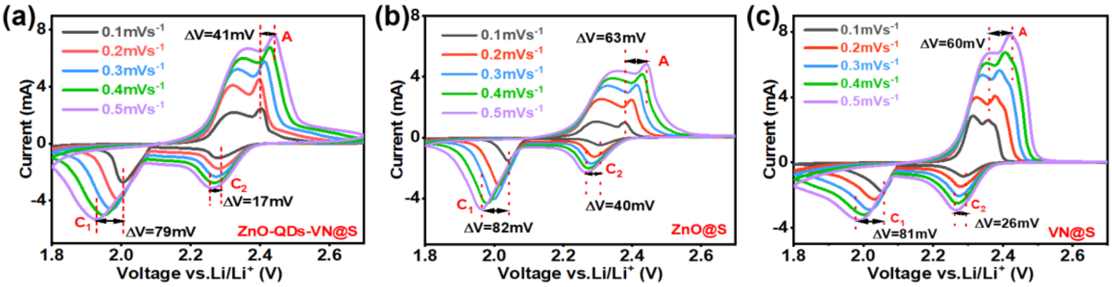

Figure S11. CV curves of ZnO-QDs-VN@S, ZnO@S, and VN@S at the rate of 0.1-0.5 mV s^{-1}.

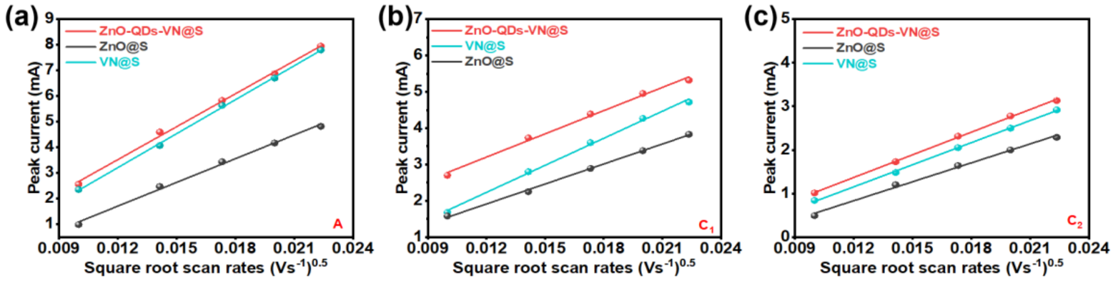

Figure S12. (a) A, (b) C_1, and (c) C_2 peak current and square root of scan rate.

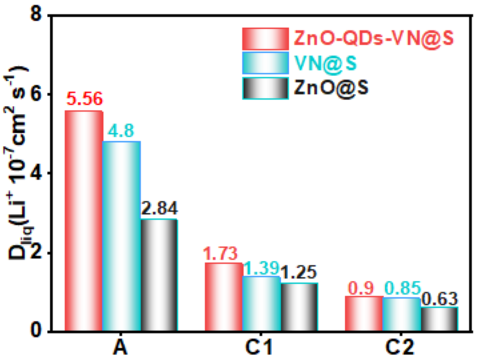

Figure S13. Li^+ diffusion coefficients calculated from CV curves for different cathodes.

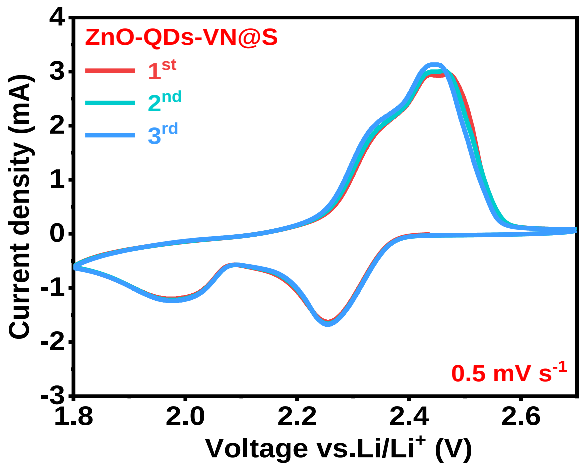

Figure S14. Three cycles of CV curves of ZnO-QDs-VN@S cathode at 0.5 mV s^{-1}.

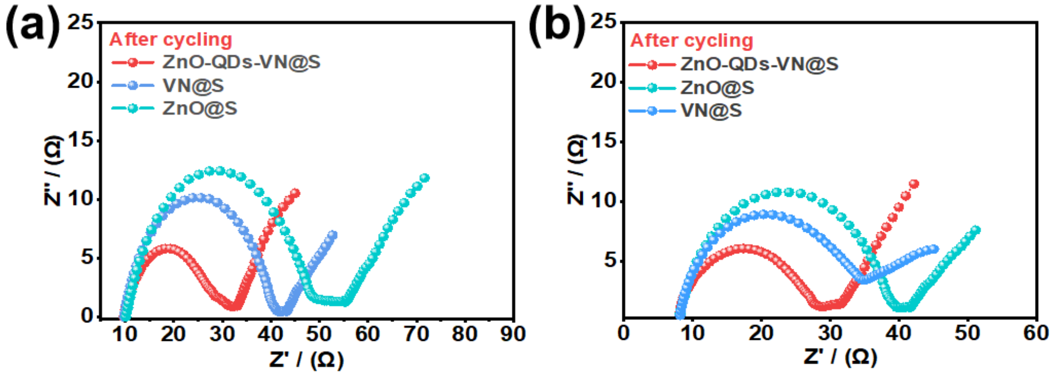

Figure S15. (a) EIS curves of ZnO-QDs-VN@S, ZnO@S, and VN@S after 100 cycles (data are mean ± 2.2 Ω from three independent experiments). (b) EIS curves of ZnO-QDs-VN@S, ZnO@S, and VN@S after 150 cycles (data are mean ± 1.8 Ω from three independent experiments).

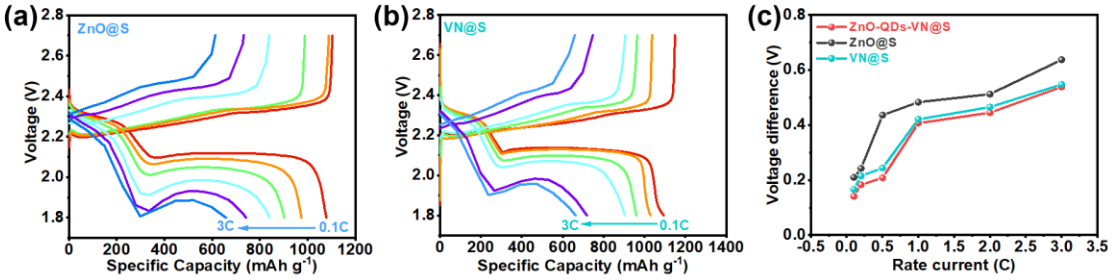

Figure S16. (a-b) Charging and discharging curves of LSBs assembled at different speeds with ZnO@S and VN@S cathodes. (c) The voltage differences of LSBs fabricated with distinct cathode materials at different rates.

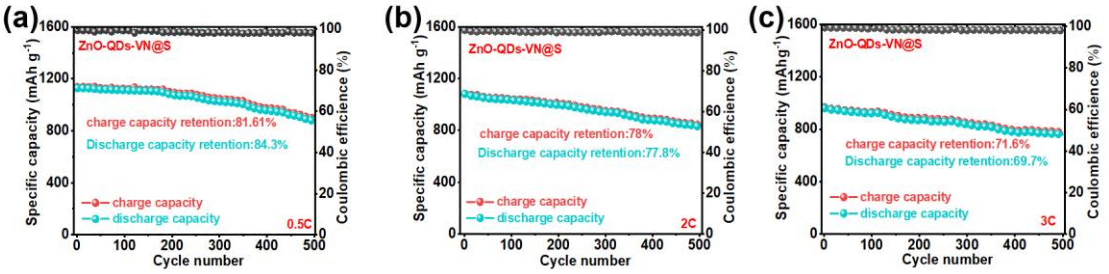

Figure S17. (a-c) Cycle performance of ZnO-QDs-VN@S at 0.5 C, 2.0 C, and 3.0 C.

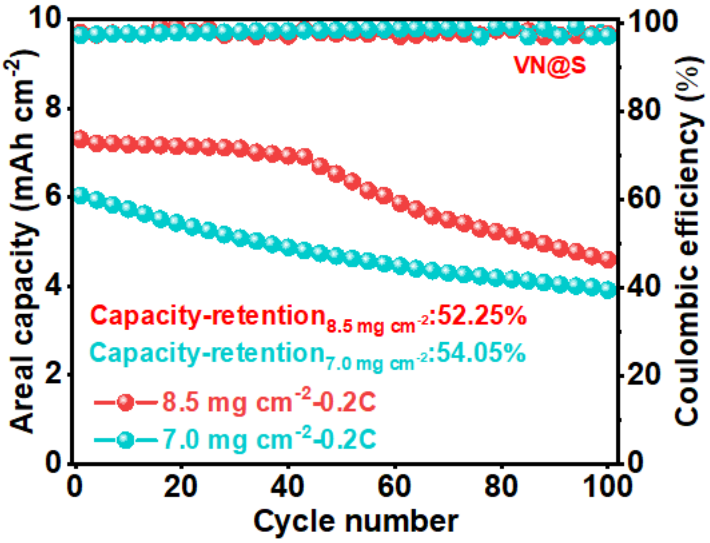

Figure S18. The areal capacity of VN@S cathodes under 0.2 C with high sulfur loading.

Supplementary Tables

Table S1. High-resolution fitting parameter table of ZnO-QDs-VN.

Name	Start Be	Peak Be	End Be	FWHE eV	Atomic (%)
C1s	297.98	284.89	279.18	1.8	60.98
N1s	409.98	397.51	392.18	1.29	17.54
O1s	544.98	530.75	525.18	3.31	9.39
V2p	539.98	514.13	507.18	2.44	10.8
Zn 2p	1051.98	1022.07	1015.18	1.73	1.3

Table S2. High-resolution fitting parameter table of VN.

Name	Start Be	Peak Be	End Be	FWHE eV	Atomic (%)
C1s	297.98	284.09	279.18	1.6	55.02
N1s	409.98	396.54	392.18	1.9	7.58
O1s	544.98	525.18	525.18	1.78	22.67
V 2p	539.98	529.58	507.18	1.83	14.73

Table S3. The discharge capacity and ratio between the two discharge platforms

Sample	Q1	Q2	Q2/Q1
**ZnO-QDs-VN@S**	**398.6**	**891.2**	**2.24**
ZnO@S	335.9	691.5	2.05
VN@S	392.7	837.2	2.13

Table S4. Comparison of the ZnO-QDs-VN@S performance with other published Works

Sample	Current rate (C)	Cycle number	Capacity (mAh g^{-1})	Capacity decay rate (per cycle)	Reference
**ZnO-QDs-VN@S**	**1 C**	**500**	**984.4**	**0.023%**	**This work**
rGO@ZnO-QDs/S	1 C	400	732.12	0.066%	S1[1]
S/MoN-VN	1 C	500	555	0.056%	S2[2]
VN/Co3ZnC@NCNT/S	1 C	500	557.6	0.064%	S3[3]
S/VN/N-rGO	1 C	100	765	0.056%	S4[4]
S/ZCCDNC	1 C	500	501.05	0.076%	S5[5]
3DOM NC@V-ZnO/S	1 C	500	785	0.043%	S6[6]
ZnO/BiOI/CNT/S	1 C	400	586.05	0.082%	S7[7]
CC/VN/Co@NCNTs/S	1 C	500	531.4	0.063%	S8[8]

Supplementary Equations

Equation S1:

Calculation of D_{Li^+} from CV curves

The diffusion process of Li^+ can be described through the Randles-Sevcik equation as follows:

$$Ip=2.69\times10^5 n^{3/2} A D^{1/2}_{Li^+} C_{Li^+} V^{1/2}$$

Where Ip indicates the peak current, n is the number of electron transfers (n=2), A means the electrode area (1.13 cm^2), C_{Li^+} represents the Li^+ concentration in the electrolyte (mol cm^{-3}), V is the scanning rate (0.1-0.5 mV s^{-1}), and D_{Li^+} shows the diffusion coefficient of Li ions (cm^2 S^{-1}).

Equation S2

Calculation of D_{Li^+} from GITT curves

The diffusion process of Li^+ can be described through the Wagner equation as follows:

$$D\approx4L^2/\pi T\times(\Delta E_{ss}/\Delta E_{T/2})^2$$

L indicates the diffusion path thickness at the electrode/electrolyte interface (cm), T represents the duration of the constant current pulse (s), ΔEss means the stable potential difference after the pulse ends (V), and $\Delta E_{T/2}$ shows the potential difference at half the pulse duration (V).

Equation S3

Calculation of internal resistance from GITT

$$\Delta R_{internal} = | \Delta V_{QOCV-CCV} | / I_{applied}$$

Where $\Delta V_{QOCV-CCV}$ is the potential difference between the closed-circuit voltage (CCV) and the quasi-open circuit voltage (QOCV), $I_{applied}$ is the constant current through the reaction, and $\Delta R_{internal}$ is the internal resistance of the battery during discharging and charging.

References

1. Jian, Z.; Zhang, S.; Guan, X.; Li, J.; Li, H.; Wang, W.; Xing, Y.; Xu, H. ZnO quantum dot-modified rGO with enhanced electrochemical performance for lithium–sulfur batteries. *RSC Advances* **2020**, *10*, 32966-32975,doi. 10.1039/D0RA04986G.
2. Ye, C.; Jiao, Y.; Jin, H.; Slattery, A.D.; Davey, K.; Wang, H.; Qiao, S.-Z. 2D MoN-VN Heterostructure To Regulate Polysulfides for Highly Efficient Lithium-Sulfur Batteries. *Angewandte Chemie International Edition* **2018**, *57*, 16703-16707,doi. https://doi.org/10.1002/anie.201810579.
3. Fu, J.; Shen, Z.; Cai, D.; Fei, B.; Zhang, C.; Wang, Y.; Chen, Q.; Zhan, H. A hierarchical VN/Co_3ZnC@NCNT composite as a multifunctional integrated host for lithium–sulfur batteries with enriched adsorption sites and accelerated conversion kinetics. *Journal of Materials Chemistry A* **2022**, *10*, 20525-20534,doi. 10.1039/D2TA06231C.
4. Li, N.; Xu, Z.; Wang, P.; Zhang, Z.; Hong, B.; Li, J.; Lai, Y. High-rate lithium-sulfur batteries enabled via vanadium nitride nanoparticle/3D porous graphene through regulating the polysulfides transformation. *Chemical Engineering Journal* **2020**, *398*, 125432,doi. https://doi.org/10.1016/j.cej.2020.125432.
5. Xu, P.; Liu, H.; Zeng, Q.; Li, X.; Li, Q.; Pei, K.; Zhang, Y.; Yu, X.; Zhang, J.; Qian, X.; et al. Yolk−Shell Nano ZnO@Co-Doped NiO with Efficient Polarization Adsorption and Catalysis Performance for Superior Lithium−Sulfur Batteries. *Small* **2021**, *17*, 2005227,doi. https://doi.org/10.1002/smll.202005227.
6. Zhao, X.; Guan, Y.; Du, X.; Liu, G.; Li, J.; Li, G. Ordered macroporous V-doped ZnO framework impregnated with microporous carbon nanocages as multifunctional sulfur reservoir in lithium-sulfur batteries. *Chemical Engineering Journal* **2022**, *431*, 134242,doi. https://doi.org/10.1016/j.cej.2021.134242.
7. Zeng, P.; Yu, H.; Chen, M.; Xiao, W.; Li, Y.; Liu, H.; Luo, J.; Peng, J.; Shao, D.; Zhou, Z.; et al. Flower-like ZnO modified with BiOI nanoparticles as adsorption/catalytic bifunctional hosts for lithium–sulfur batteries. *Journal of Energy Chemistry* **2020**, *51*, 21-29,doi. https://doi.org/10.1016/j.jechem.2020.03.040.
8. Cai, D.; Zhuang, Y.; Fei, B.; Zhang, C.; Wang, Y.; Chen, Q.; Zhan, H. Self-supported VN arrays coupled with N-doped carbon nanotubes embedded with Co nanoparticles as a multifunctional sulfur host for lithium-sulfur batteries. *Chemical Engineering Journal* **2022**, *430*, 132931,doi. https://doi.org/10.1016/j.cej.2021.132931.
